# Characterization of Coelomic Fluid Cell Types in the Starfish *Marthasterias glacialis* Using a Flow Cytometry/Imaging Combined Approach

**DOI:** 10.3389/fimmu.2021.641664

**Published:** 2021-03-18

**Authors:** Claúdia Andrade, Bárbara Oliveira, Silvia Guatelli, Pedro Martinez, Beatriz Simões, Claúdia Bispo, Cinzia Ferrario, Francesco Bonasoro, José Rino, Michela Sugni, Rui Gardner, Rita Zilhão, Ana Varela Coelho

**Affiliations:** ^1^CEDOC, Chronic Diseases Research Centre, NOVA Medical School, Faculdade de Ciências Medicas, Universidade NOVA de Lisboa, Lisboa, Portugal; ^2^Flow Cytometry SRL, Instituto Gulbenkian Ciencia, Oeiras, Portugal; ^3^Instituto de Tecnologia Química e Biológica António Xavier, Universidade Nova de Lisboa, Oeiras, Portugal; ^4^GAIA 2050 Center, Department of Environmental Science and Policy, University of Milan, Milan, Italy; ^5^Departament de Genètica, Microbiologia i Estadística, Universitat de Barcelona, Barcelona, Spain; ^6^ICREA (Institut Català de Recerca i Estudis Avancats), Barcelona, Spain; ^7^Center for Complexity and Biosystems, Department of Physics, University of Milan, Milan, Italy; ^8^Instituto de Medicina Molecular João Lobo Antunes, Faculdade de Medicina, Universidade de Lisboa, Lisboa, Portugal; ^9^Departamento de Biologia Vegetal, Centro de Ecologia, Evolução e Alterações Ambientais, Faculdade de Ciências, Universidade de Lisboa, Lisbon, Portugal

**Keywords:** coelomocytes, *Marthasterias glacialis*, echinoderms, flow cytometry and imaging flow cytometry, fluorescence and transmission electron microscopy

## Abstract

Coelomocytes is the generic name for a collection of cellular morphotypes, present in many coelomate animals, and highly variable among echinoderm classes. The roles attributed to the major types of these free circulating cells present in the coelomic fluid of echinoderms include immune response, phagocytic digestion and clotting. Our main aim in this study was to characterize coelomocytes found in the coelomic fluid of *Marthasterias glacialis* (class Asteroidea) by using a combination of flow cytometry (FC), imaging flow cytometry (IFC) and fluorescence plus transmission electron microscopy (TEM). Two coelomocyte populations (P1 and P2) identified through flow cytometry were subsequently studied in terms of abundance, morphology, ultrastructure, cell viability and cell cycle profiles. Ultrastructurally, P2 diploid cells were present as two main morphotypes, similar to phagocytes and vertebrate thrombocytes, whereas the smaller P1 cellular population was characterized by low mitotic activity, a relatively undifferentiated cytotype and a high nucleus/cytoplasm ratio. In the present study we could not rule out possible similarities between haploid P1 cells and stem-cell types in other animals. Additionally, we report the presence of two other morphotypes in P2 that could only be detected by fluorescence microscopy, as well as a morphotype revealed *via* combined microscopy/FC. This integrative experimental workflow combined cells physical separation with different microscopic image capture technologies, enabling us to better tackle the characterization of the heterogeneous composition of coelomocytes populations.

## Introduction

*Marthasterias glacialis* (Linnaeus, 1758), commonly known as the spiny starfish, is a member of the class Asteroidea (phylum Echinodermata). Members of this class (i.e., Asteroids) are known for their remarkable regenerative abilities (1, 2), which allow the animals to restore and regrow lost body parts after injury, providing an ecological advantage in environments shared with predators. The regenerative process involves the mobilization of many cell types to the area of injury to perform two major processes: 1) monitor and clearing environmental pathogens that have gained sudden access to internal fluids, and 2) healing the wound and subsequently rebuilding the missing structures. It has been known for some time that coelomocytes, the circulating cells present in the coelomic cavities, play a critical role in all these processes (3). Coelomocytes are mostly known as effectors of the immune response and are thus key contributors to the monitoring of pathogens and restoring of missing tissues (4). How these processes occur remains a matter of debate, due in great part to the still limited knowledge of coelomic fluid composition and of the mechanistic/molecular basis of coelomocytes functions.

In echinoderms, the coelomic fluid completely fills the coelomic spaces of the body, including the perivisceral coelomic cavities, the water vascular system and the perihemal systems (5–7). Coelomocytes perform diverse immune functions, such as cellular clots formation, phagocytosis, encapsulation, and clearance of bacteria and other foreign materials (7, 8). After starfish experience traumatic injury or autotomy, coelomocytes rapidly aggregate in the damaged area, forming a clot that seals the inner environment from the external milieu, thus preventing the loss of coelomic fluid and ensuring hemostasis (8, 9).

Recently, the pharynx and axial organs were proposed as the echinoid analogues of hematopoietic organs (10). Despite such hypotheses, the specific tissue where starfish coelomocytes originate remains unknown. Researchers have proposed that these cells originate in the axial organ (11), the Tiedemann’s bodies (12) or the perivisceral coelomic epithelium (CE) (13, 14). This final view is currently considered the most plausible, supported by several experimental results that show release of cells from the CE in response to injury or to exposure to foreign particles (13, 15, 16).

While the main activities of the coelomocytes are known (most relate to immune response as stated above), the number, types and specific physiological contribution of each morphotype are still poorly understood. Indeed, due to the diversity in morphological descriptions offered in the literature, use of different names for similar cellular phenotypes, changes in experimental outcomes arising from alternative coelomocyte’s handling protocols and the diversity of characterization methods, these circulating cells are subjected to various classification schemes and therefore cannot yet be systematically identified. The main consequence of this is an intrinsic difficulty in homologizing cell types within and across different echinoderm classes. For example, Smith et al. (7) identified only three categories of coelomocytes in sea urchins (phagocytes, spherule cells or amoebocytes, and vibratile cells). Meanwhile, using May-Grunwald/Giemsa staining, Taguchi et al. (17) tentatively classified coelomocytes of the sea cucumber *Apostichopus japonicus* into up to twelve types, including several granulocytes (17). In the starfish *Asterias rubens*, Gorshkov et al. (18) detected just two types of cells and termed them “mature” and “immature” coelomocytes. In our model starfish species, *M. glacialis*, Franco (19) used light microscopy to distinguish the presence of four types of coelomocytes: spherule cells, vibratile cells, amoebocytes and phagocytes (the final type showing petaloid or filopodial alternate morphologies). Given the range and diversity of reported morphologies, it is nowadays unanimously accepted that the types and characteristics of coelomocytes simply vary among the five echinoderm classes (3). In fact, an agreement on the classification of the intra-class (*i.e.* Asteroidea) coelomocytes has not yet been reached, likely also due to a combination of the intrinsic heterogeneity of morphotypes, poor taxonomic sampling and, as already mentioned variability in experimental protocols.

In order to contribute to a greater understanding of the cellular components present in the coelomic fluid of an asteroid, we carried out a comprehensive analysis and the characterization of coelomocytes of the starfish *M. glacialis*. We used IFC and fluorescence microscopy to implement optimized experimental protocols for the study of many different coelomocyte characteristics, such as cell viability, cell cycle and cell morphology. Our results allowed clarifying the cytological diversity of coelomocytes present in Asteroidea. Whenever the current knowledge allowed, we incorporated an inter-class perspective in our discussion.

## Materials and Methods

### Animal Collection and Maintenance

Adult specimens of both sexes of the starfish *M. glacialis* were collected at low tide on the west coast of Portugal (Estoril, Cascais). The animals were transferred to the Vasco da Gama Aquarium (Dafundo, Oeiras) where they were kept in open-circuit tanks with re-circulating seawater at a temperature of 15°C and a salinity of 33‰. They were fed *ad libitum* with a diet of mussels collected from the same site. All specimens were maintained in the same conditions throughout the whole experimental procedure to control for the influence of abiotic factors (e.g., changes in salinity or temperature).

### Coelomic Fluid Collection

To minimize contamination, coelomic fluid was collected with a 21-gauge-butterfly needle and transferred directly to a Falcon tube kept on ice. The needle was inserted in the aboral side no further than 1 cm from the distal tip of the starfish arm to avoid the disruption of internal organs (*i.e.*, pyloric caeca and gonads) while coelomic fluid was collected. Coelomic fluid samples were transported on ice for all downstream experiments. To minimize coelomocyte aggregation, all samples were carefully re-suspended with a micropipette. Moreover, to avoid over-manipulation of the cells, labeling dyes were added *in situ*, directly to the collected coelomic fluid.

### Flow Cytometry

We performed preliminary filtration through a 40 μm mesh prior to flow cytometry (FC) analysis to avoid capillary clotting. We analyzed samples using CyAn ADP^™^ (Beckman Coulter) and LSRFortessa™ (BD Biosciences) flow cytometers. Lasers and filters λ, respectively, for flow cytometers were for DRAQ5 642; 665/20 BP (Cyan ADP™) and 633 nm; 660/20 BP (LSRFortessa™), for DAPI 405 nm; 450/50 BP (Cyan ADP™) and for PI 488 nm; 670/30 BP (LSRFortessa™). We further analyzed the data using FlowLogic (Inivai Technologies) and FlowJo software (version 10.7, Becton, Dickinson & Company).

### Imaging Flow Cytometry

Just before each Imaging Flow Cytometry (IFC) assay, we filtered 200 μL of coelomic fluid (average number of cells: 90,000) to remove cellular aggregates. The vacuolar membranes and nucleus were each stained at RT for 5 minutes, respectively, with 0.5 μL of FM4-64 (Molecular Probes, #T3166; vacuolar) at a concentration of 5 μg/mL and 1.0 μL of DRAQ5 (Thermo Fisher Scientific, #65-0880-92; DNA) at a concentration of 5 mg/mL. In a series of control assays, we confirmed that increasing the staining time did not increase the signal in labeled cells.

We acquired coelomocyte images using the INSPIRE software of the ImageStreamX Mark II imaging flow cytometer (Luminex Corporation, Austin, TX) at the Instituto de Medicina Molecular João Lobo Antunes, Faculdade de Medicina, Universidade de Lisboa, Portugal. Cells were imaged at 60x magnification using a 488 nm laser at 15 mW for FM4-64 excitation with a detection window of 642-745 nm (channel 5) and a 642 nm laser at 150 mW for DRAQ5 excitation with a detection window of 642-745 nm (channel 11). We obtained brightfield images *via* channels 1 and 9. Following the gating strategy described in our Results section, we analyzed IFC data using the IDEAS (v6.2) software.

### Cell Viability Assay

To evaluate cell viability, fresh cells were stained with DAPI (Molecular Probes) and DRAQ5 (Molecular Probes) and then assayed using a CyAn ADP High-Performance Flow Cytometer Analyser. To check for consistency, we tested other DNA dyes for dead/permeabilized cells: propidium iodide (PI; Thermo Fisher), DRAQ7 (Thermo Fisher) and 7-ADD (Thermo Fisher). The final dye concentrations used were 0.5 μg/mL, 0.05 μM, 1 μg/mL, 0.03 μM and 0.5 μg/mL, respectively.

### Cell Cycle Analysis

To study cell cycle progression, an initial cell permeabilization step is necessary; in the present study, we implemented a protocol adapted from (20). Cells from the coelomic fluid were collected by centrifugation at 1000*g* for 5 minutes at 4°C. Pelleted cells were washed once in 3.5% (w/v) Artificial Sea Water (ASW; prepared from Sea Salts, Sigma). Cold 70% (v/v) ethanol was added in individual drops to the pellet during slow vortexing. After, at the latest, 24 hours post collection, the cells were rinsed twice in ASW and incubated for 1 hour in a solution: 5% RNAse1 at 100 μg/mL in 85% ASW and 10% PI at 1 mg/mL. Samples were kept protected from light and transferred to a BD LSRFortessa flow cytometer (laser beam λ set at 488 nm and filter set at λ 670/30 nm). Before running the samples stained for DNA content, we verified the cytometer’s linearity, resolution and doublet discrimination capability using a DNA QC kit from Becton Dickinson. We carried out our data analysis using the Dean Jett Fox algorithm of the FlowJo software package (version 10.7, Becton, Dickinson & Company).

### Fluorescence Microscopy

In order to analyze the morphology of cells present in the coelomic fluid, we used 199 μL from the cell suspension pellet and followed a staining protocol with FM4-64 (Molecular Probes), similar to that used for IFC with the addition of DAPI. We added 1 μL of FM 4-64 directly to the coelomic fluid before incubating it for 2 minutes. Ten μL of this suspension were deposited for each well of a microscope slide (Marienfeld Superior) before 1 μL of DAPI was added and the mixture homogenized. We acquired microscopy images using a LEICA DM 6000B upright microscope equipped with an Andor iXon 885 EMCCD camera and controlled *via* MetaMorph software (version 5.8), using a 100x/1.4 oil immersion objective plus a 1.6x optovar (pixel size: 0.05 μm), fluorescence filter sets for DAPI (excitation: 340–380 nm; dichroic 400 nm; emission: 450–490 nm) and FM 4-64 (excitation: 540–580 nm; dichroic 595 nm; emission: 608–683 nm), and phase contrast optics.

### Ultrastructural Analysis

Coelomic fluid was collected with a needle as above described and dropped directly into the fixative solution (2% glutaraldehyde and 1.2% NaCl in 0.1 M sodium cacodylate buffer at 4°C), where it was left for at least 2 hours. Throughout the protocol, the fixative solution was changed through centrifugation of cells at 400-600*g* for 5 minutes and subsequent re-suspension of the pellet. Cells were washed a few times in a 0.1 M sodium cacodylate buffer and left in the same buffer overnight at 4°C, post-fixed with 1% osmium tetroxide in 0.1 M sodium cacodylate buffer for 2 hours at RT and washed several times with distilled water. The samples were then left in 2% uranyl acetate in 25% ethanol for 2 hours at RT before being dehydrated in a series of increasingly concentrated ethanol solutions (25%, 50%, 70%, 90%, 95%, 100%), washed in propylene oxide, and washed in a gradual series of solutions containing epoxy resin (Epon 812-Araldite) and propylene oxide mixes of different proportions (1:3, 1:1, 3:1). Finally, these cells were embedded in fresh resin (Epon 812-Araldite).

Using a glass or a diamond knife, we obtained ultrathin sections (between 70-90 nm in thickness) from the cellular pellets using a Reichert-Jung Ultracut E microtome. We then mounted the sections on copper grids (150 and 400 mesh) and stained them with a solution of 1% uranyl acetate followed by a lead citrate solution. These ultrathin sections were then observed and photographed under a CM10 Philips transmission electron microscope (TEM) equipped with a Morada Soft Imaging System digital camera operated with Item Software.

## Results

### Coelomocyte Fractionation Using Flow Cytometry

**Figure 1:**
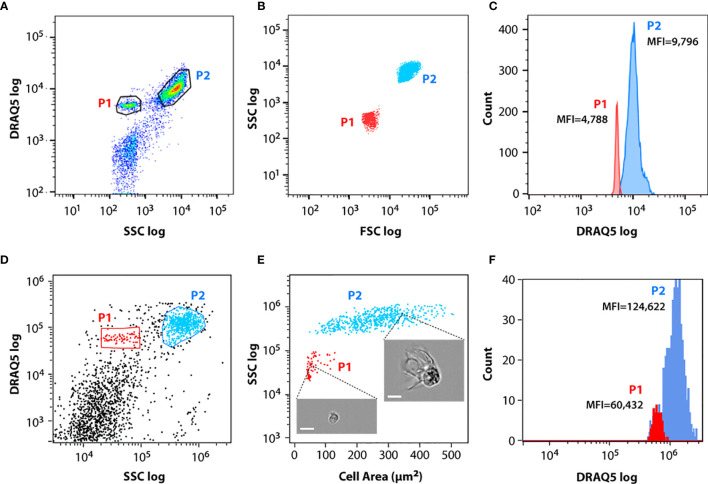
Flow cytometry (FC) and imaging flow cytometry (IFC) analysis of circulating coelomocytes. Coelomocytes were stained with DRAQ5. **(A)** Representative FC dot plot of coelomocytes with cell populations gated. **(B)** Overlap of FC dot plots showing representative forward and side scatter properties of P1 (red) and P2 (blue) cell populations. **(C)** Overlap of P1 (red) and P2 (blue) FC cell populations histograms showing their median fluorescence intensity (MFI) in the DRAQ5 (660/20 BP) channel. **(D)** Representative IFC dot plot of coelomocytes with cell populations gated. **(E)** Overlap of IFC dot plots showing representative area and side scatter properties of P1 (red) and P2 (blue) cell populations. Insets show two examples of cells imaged in each population; scale bar: 7 μm. **(F)** Overlap of P1 (red) and P2 (blue) IFC cell populations histograms showing their median fluorescence intensity (MFI) in the DRAQ5 (642-745 nm) channel.

The isolated coelomic fluid from different animals was transferred immediately to our cytometry facility. The flow cytometry analysis of the cells isolated from *M. glacialis* fluid revealed two clearly distinguishable cell populations, termed P1 and P2 in this study (**Figure 1A**). These two populations differed mainly in the value of their light scattering, with P2 cells showing higher forward scatter (FSC) and side (SSC) scatter values than P1 cells (**Figure 1B**), likely a direct consequence of P2 cells´ more complex/structured surface (including particulate material, such as inclusions or granules) and richer internal cyto-architecture. P2 represented 60-70% of the total coelomocytes (**Table S1**) and a maximum of 20% of the cells were osmotically compromised, displaying lower DRAQ5 incorporation/fluorescence values (**Figure 1A**). Direct comparison between DRAQ5 intensity values (Mean Fluorescence Intensity or MFI) for both populations showed that P2 cells incorporated approximately twice the amount of DRAQ5 than P1 cells (**Figure 1**).

The same labeled cell suspensions were analyzed by IFC. Single cells were identified using the area and aspect-ratio features of the brightfield channel. We selected only focused cell images for further analysis, using the Gradient RMS function in IDEAS to distinguish between focused (high gradient RMS value) and unfocused images (low gradient RMS value). P1 and P2 cells were best identified using the DRAQ5 and SSC channels (**Figure 1D**). P2 cells displayed a large dispersion in area values, ranging from 100 to 500 μm^2^, while P1 cells were smaller, with areas values ranging from 50 to 130 μm^2^ (**Figure 1E**). A histogram plot of DRAQ5 staining for both populations showed that the median fluorescence intensity (MFI) of DRAQ5 measured by IFC for P2 was approximately twice the MFI value measured for P1 (**Figure 1F**), consistent with the results obtained *via* conventional flow cytometry (**Figure 1C**), thus confirming that P1 and P2 represented the same respective cell populations when analyzed using the two FC techniques.

Due to their clotting functions, coelomocytes tended to aggregate during the collection process. As a consequence, we cannot exclude the possibility that a fraction of coelomic cells were retained during the filtration step (through a 40 μm nylon cell strainer step) that was necessarily performed prior to the flow cytometry analysis. Moreover, the inclusion of anticoagulant solutions, such as 1.9% sodium citrate (1:1) (21, 22) and isotonic 1:1 anticoagulant buffer, in the suspension (0.5 M NaCl, 5 mM MgCl_2_, 20 mM HEPES and 1 mM EGTA pH=7.5) (23) produced the unwanted effect of extensive cell loss and compromised morphological integrity in the remaining cells (see **Figure S1**). Resuspension and subsequent filtering of cells were implemented to avoid cell aggregation. This decision followed Matranga et al. (24), who specifically recommended a better way to standardize findings across animal samples: “to suggest a nomenclature that reflects the actual morphology of the cells requires the immediate observation of fresh and alive cells, just taken from the sea urchin without any addition of anti-coagulant solutions”.

### Evaluation of Cell Viability

**Figure 2:**
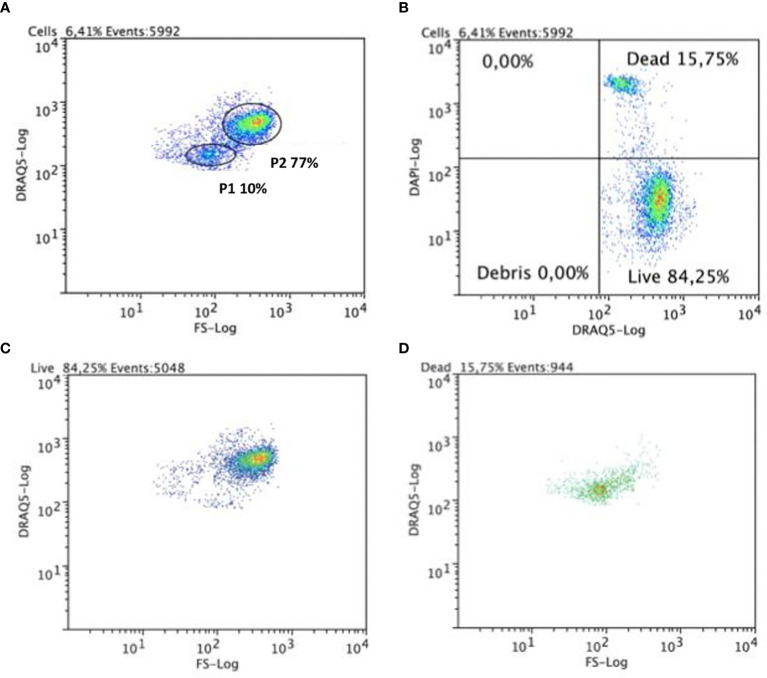
Flow cytometric analysis of circulating coelomocytes viability. Cells were stained with DRAQ5 and DAPI. **(A)** Representative dot plot with P1 and P2 cell populations gated. **(B)** A quadrant plot of coelomocytes showing DAPI+ DRAQ5+ (dead) and DRAQ5+ DAPI- (alive) coelomocytes. **(C)** A dot plot showing that in the living cells quadrant, these belong mainly to the P2 cell population. **(D)** A dot plot showing that cells in the dead cells’ quadrant belong mainly to P1 cell population.

Samples of collected coelomic fluid consistently contained some cellular debris (detected by FC), material which could interfere with the correct display of coelomocyte populations in cytometry. We used DRAQ5, a lipophilic and membrane-permeable DNA dye, for the detection of live cells (25), which allowed us to directly discriminate coelomocytes from cellular debris. Additionally, and in order to better evaluate coelomocyte population viability, we employed the use of compatible exclusion dyes that is, reagents excluded by healthy cells (due to the integrity of their plasma membranes) that also do not interfere with DRAQ5 fluorescence or compete for its DNA binding activity). DAPI co-staining fulfilled this criterion, revealing that the P1 cells incorporated it readily, whereas the P2 cells seemed more refractory and thus represented a population with relatively few DAPI-positive cells in our assays (**Figure 2**). One possible explanation for the labeling patterns we observed is that, on average, P1 cells exhibited a relatively low degree of viability, whereas the majority of P2 cells were normally viable. Although the low P1 viability remained stable when kept outside the organism over a period of one day, the viability of P2 seemed to decrease sharply during this same period. To test if cellular permeability to DAPI was due only to a loss of viability (26), we also tested alternative exclusion dyes (PI, DRAQ7 and 7-ADD). In all cases, the results were nearly identical to those obtained with DAPI (data not shown).

After sorting, we excluded preliminary fluorescence microscopy tests to ascertain the purity of the samples and determine preliminary characterization of the cellular morphologies of the two populations (data not shown). P1 cells were less abundant than P2 cells, measured an average of 4 μm in diameter, and showed a spherical morphology. Meanwhile, P2 cells presented a diversity of morphotypes with diameters ranging from 3 to 10 folds those determined for the P1 cells. Notably, the diversity in morphology observed in P2 cells could be attributed to particularities of the sorting process. In fact, under higher magnification fluorescence microscopy, using DAPI and FM4-64 to specifically label DNA and cell membranes, respectively, it was possible to confirm that, regardless of assigned population, most sorted-cells underwent quick lysis, leaving the total number of intact cells remarkably low. These effects were also observed 4 hours after collection and again when cells were centrifuged and fixed with ethanol. In both cases, the impact of experimental manipulations was not homogenous across the different cell types. Based on this observation, no further cell sorting was performed before microscopy with the aim of ensuring that a morphological characterization could be performed without significant data distortion.

### Analysis of the Cell Cycle

**Figure 3:**
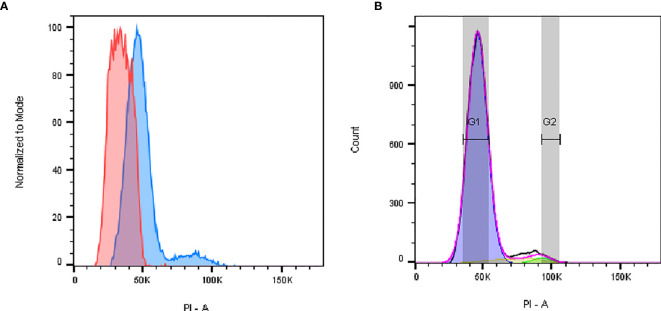
Flow cytometric cell cycle histograms. Cells were fixed with 70% ethanol and stained with PI. **(A)** Representative histogram of P1 (red) and P2 (blue) singlets in the cell cycle, showing that for P1 most of the cells are in G0/G1 phase. **(B)** P2 cell distribution in G0/G1 (blue, 92%), S (yellow, 5%), or G2/M (green, 2%) phases was determined using the Dean Jett Fox fitting algorithm of FlowJo software (version 10.7), after excluding cell debris and aggregates. PI fluorescence is proportional to DNA content.

To study the cell cycle profile, and to determine the fraction of cells undergoing the individual phases, we loaded cells into the cytometer after fixation and an extensive DNA labeling period with PI to ensure proportionality. Data were acquired in linear mode in order to detect n-fold differences in DNA content (**Figure 3A**). We detected that the P2 fraction of cells was concentrated in the first phase of the cell cycle G0G1 and had a 2n DNA content (**Figure 3B**). P2 cells also seemed to go through a regular cell cycle, with fractions of cells observed in all phases. On the other hand, a unique histogram peak was observed for P1 cells indicating that all were likely in a unique phase of the cycle (**Figure 3A**). The simple/single profile of the P1 cells can be ascribed to either a quiescent state (known as G0 phase) or to a terminally differentiated one. In contrast to P2, P1 cells did not seem to undergo division. As shown below in the IFC results, each cell populations unveiled a heterogeneous set of morphotypes. This fact could explain the high variability of the average DNA content, particularly in the P1 cells, as reflected by the width of the P1 peak shown in the histogram of **Figure 3A**. In particular for P1, we observed by fluorescence microscopy more extensive morphological degradation of some cells (see below). However, a sub-G1 peak was not detected, indicating the loss of DNA fragments by apoptotic cells; a range of cells was detected with a fractional ploidy between n and 2n, suggesting DNA leakage from the ethanol-fixed P1 cells (27). Consequently, we cannot rule out the possible presence of P1 cells with a DNA content of n (haploid).

### Coupling FC With Imaging for Coelomocyte Populations Fractionation and Morphological Characterization

**Figure 4:**
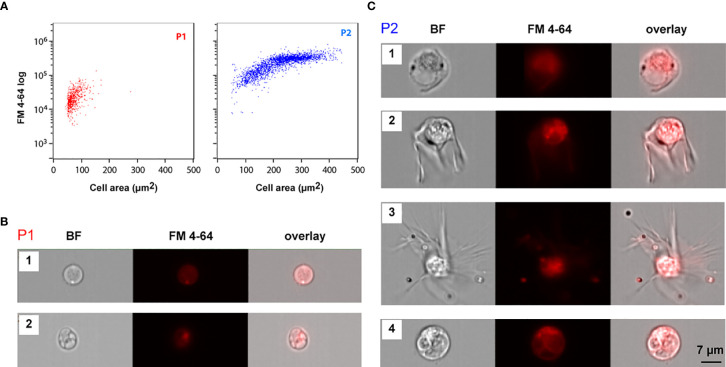
Imaging flow cytometry morphologic analysis of circulating coelomocytes. **(A)** IFC dot plots showing cell area and FM 4-64 intensity values of P1 (red) and P2 (blue) cell populations. **(B, C)** Brightfield (BF) and FM4-64 fluorescence IFC images for the several morphotypes detected within each coelomocytes population, P1 **(B)** and P2 **(C)**. Coelomocytes membranes were stained with FM 4-64 (red). B1. P1 cells displaying a less heterogenous brightfield image with the nucleus occupying the majority of the cell area (**Figure 5A3**) B2. P1 cells with a heavily granulated cytoplasm displaying a smaller nucleus to cell ratio (**Figures 5A1, A2**); C1. P2 regular; C2. P2 petaloid C3. P2 filopodial coelomocytes; C4. Big granulated cell.

IFC assays were performed using stained coelomocyte suspensions, prepared by adding 0.5 μL of FM 4-64 to 200 μL of a filtered coelomocyte suspension sample. As before, only focused images from single cells were selected for further analysis. The same P1 and P2 populations were identified by cell area and SSC (**Figure 1E**). We collected a larger number of cells in these assays to fully characterize their morphology. Whereas P1 corresponded to smaller cells (average cell area = 72 ± 21 μm^2^) with lower FM 4-64 intensity, P2 corresponded to larger cells (average cell area = 226 ± 78 μm^2^) with higher FM 4-64 intensity values (**Figure 4A**). Notice that P2 cells were also more heterogeneous, displaying higher variation in cell size (minimum area = 50 μm^2^; maximum area: 443 μm^2^). In opposition to what has been described in *Asterias forbesi*, where only phagocytes were identified (3), several cell morphologies and sizes were distinguished in the coelomic fluid of *M. glacialis*. The single cell images obtained by IFC showed that P1 cells were mostly round with varying nuclei size (**Figure 4B**), whereas P2 cells represented a mix of cell morphologies: regular, petaloid, filopodial and big granulated cells (**Figure 4C**).

**Figure 5:**
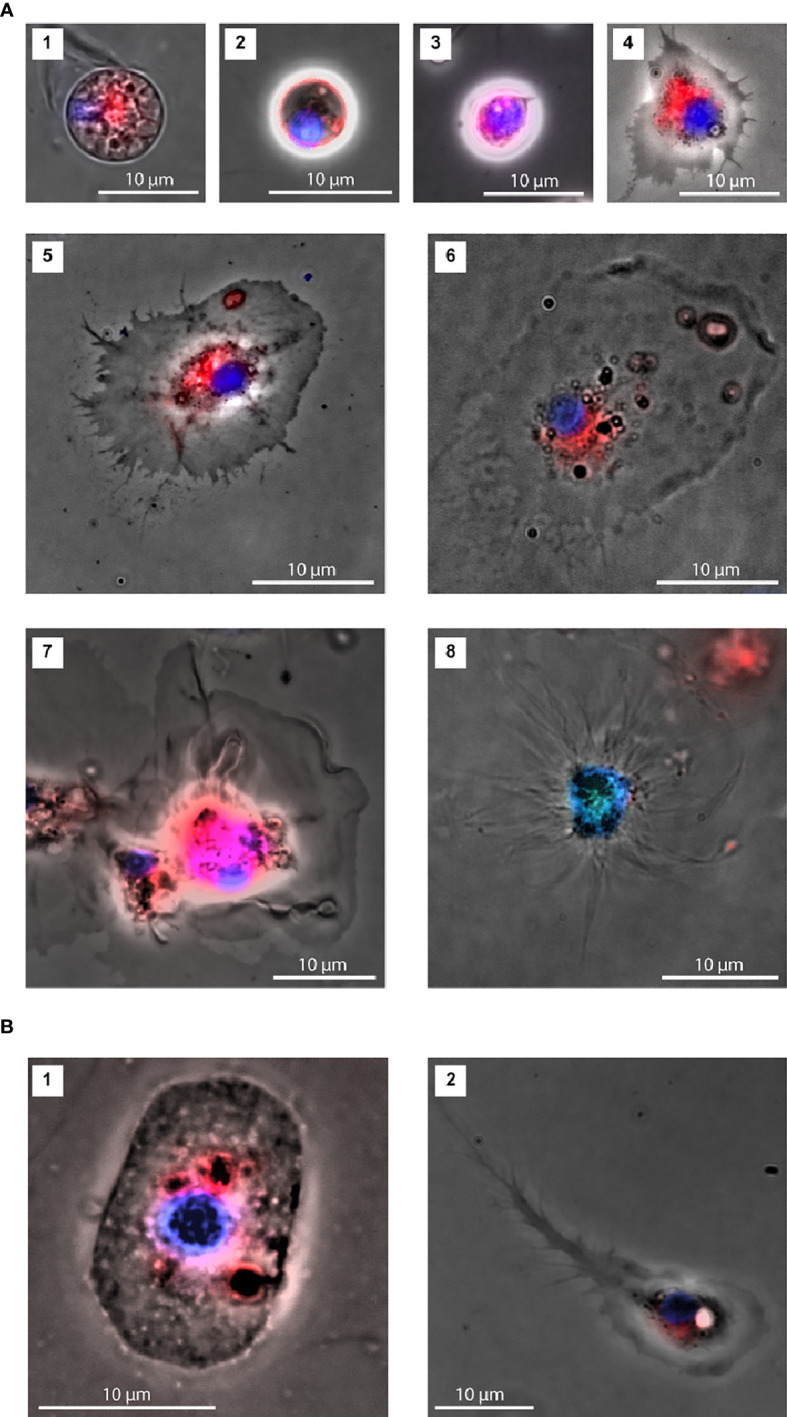
Characterization of coelomocytes morphologies by fluorescence microscopy. **(A)** Two morphotypes were identified within the P1 cell population: cells with a lower nucleus to cytoplasm ratio (**1**; area: 78 μm^2^ and **2**. area: 26 μm^2^) and cells with a nucleus occupying most of the cell space (**3**; area: 39 μm^2^). Three morphotypes were observed within the P2 cell population: Regular (**4**; area: 130 μm^2^, **5**; area: 354 μm^2^ and **6**; area: 430 μm^2^), Petaloid (**7**; area: 561 μm^2^) and Filopodial (**8**; area: 463 μm^2^). **(B)** Representatives of novel P2 cell morphologies (**1**; area: 167 μm^2^ and **2**. area: 171 μm^2^).

**Figure 6:**
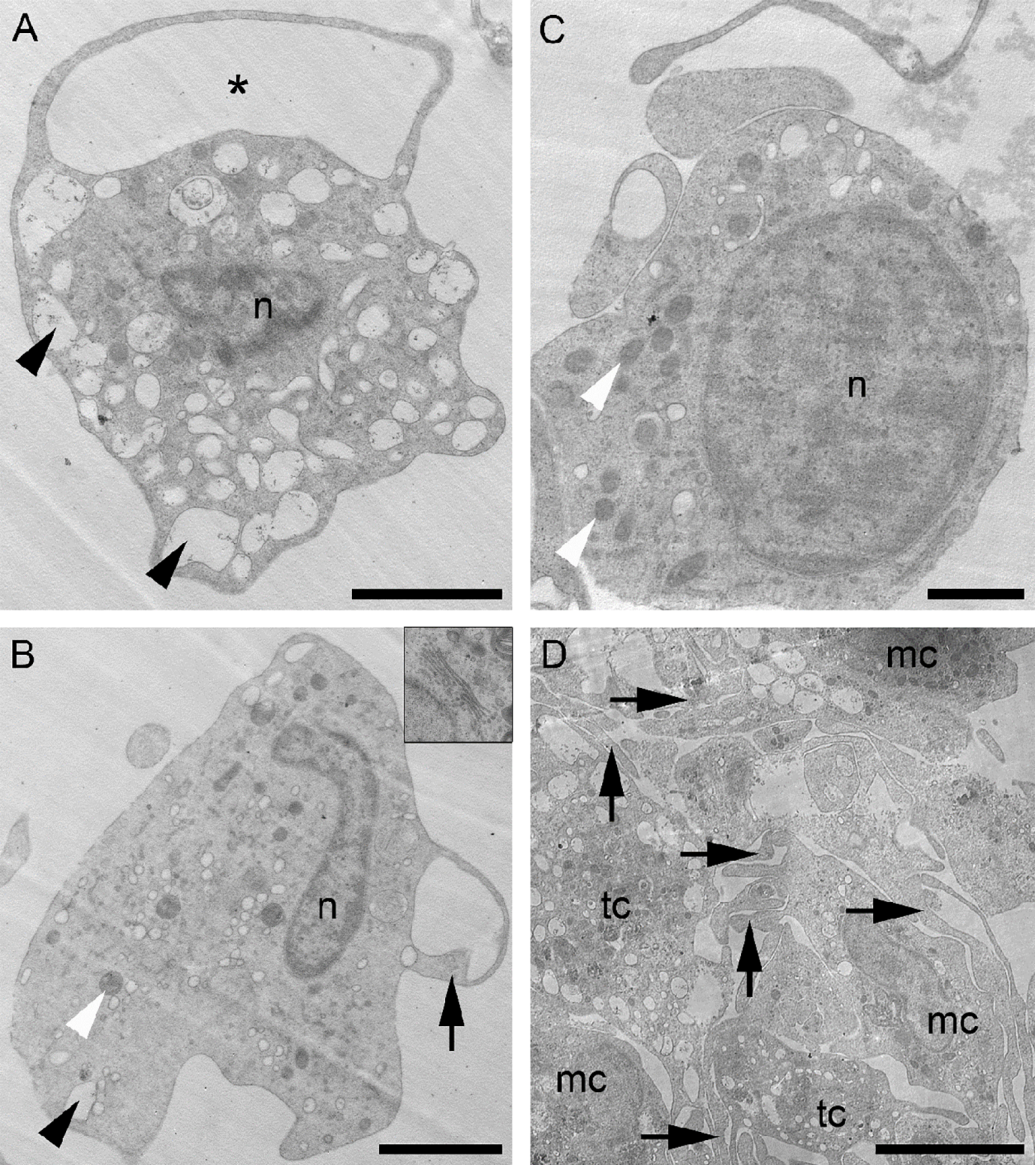
Coelomocytes morphology by transmission electron microscopy. **(A)** Thrombocyte-like cell (tc) full of roundish electron-lucent vesicles (arrowheads) and with a cell process forming a loop (asterisk). **(B)** Macrophage-like cell (mc) showing few electron-lucent vesicles (black arrowhead), mitochondria (white arrowhead) and few short cytoplasmic extensions (arrow). A well-developed Golgi apparatus is visible in the top right inset. **(C)** Slightly undifferentiated cell with a high nucleus/cytoplasm ratio and several mitochondria (white arrowheads). **(D)** Aggregate of coelomocytes: both tc and mc possess cytoplasmic processes intermingled with each other (arrows). mc, macrophage-like cell; n, nucleus; tc, thrombocyte-like cell. Scale bars: A, B=2 µm; C=0.5 µm; D=5 µm.

These cell morphologies clearly correspond to the principal cell types we observed using high resolution fluorescence microscopy (**Figure 5**) and electron microscopy (**Figure 6**), as described below in this manuscript. Though some morphological differences could be expected depending on whether the cells were in a floating or adhesive state, those differences were not observed when comparing IFC and fluorescence microscopy images. In our approach, cells were observed using slides with wells and thus likely remained in the same floating state, not contributing to variations in their morphologies. Combined IFC/microscopy results allowed to overcome the difficulties with morphological characterization of FC populations due to the coelomocyte susceptibility to sorting. This methodology provided us extensive cellular information and can eventually be used to follow changes within these populations as triggered by a diversity of environmental or physiological challenges.

### Characterization of Cell Types by Fluorescence Microscopy

Fluorescence microscopy assays were performed using coelomocyte suspensions prepared as described for IFC, after adjusting the dye concentrations. Under the experimental conditions used for the fluorescence microscopy assays, we identified two main morphotypes, both spherical, within the smaller cell population (P1). One cell type displayed a smaller nucleus-to-cytoplasm ratio, where the cytoplasm was heavily granulated and surrounded by an uninterrupted bright red membrane (P1S, **Figures 5A1, 2**). Nuclei of the second type occupied most of the cell space and showed a less heterogeneous bright field image (P1L, **Figure 5A3**).

We observed diverse P2 morphotypes, namely with regular (**Figures 5A4, 5****,** and **6**), petaloid (**Figure 5A7**) or filopodial shapes (**Figure 5A8**). The regular morphotype, also referred to as polygonal, exhibited some vesicles, mainly around the nucleus, and a spot of red staining by FM4-64 (membrane staining dye) frequently observed in the same cellular region (**Figures 5A4, 5****,** and **6**). The other two morphotypes presented clearer cytoplasm. In addition to these P2 cell types, we observed three other morphotypes in our *M. glacialis* samples that have not been previously described in the literature. One such morphotype (**Figure 5B1**) was roundish to oval in shape (~ 10 μm x 15 μm), surrounded by a membrane with irregularly distributed bright dots. Its nucleus had a round shape, was located in the center of the cell, and was usually associated with nearby dark membranous structures colored by FM4-64, the remainder of the cell was colorless and typically granulated. **Figure 5B2** shows a representative of the second new morphotype we observed. This one displayed some similarities with the polygonal phagocytes, though in this case bore a concentrated granular region that included the nucleus, surrounded by a homogenous cytoplasm extending into a notably elongated filopodium (typically 20-30 μm) terminally subdivided into a series of thin filopodia. Interestingly, only through the introduction of IFC we were able to detect a third potentially new spherical-cell morphotype in P2 that exhibited heterogeneous cytoplasm and nuclei (**Figure 4C4**). In this cell type FM4-64 staining seemed to highlight the heterogeneities of the cytoplasm.

### Coelomocyte Ultrastructure

Transmission electron microscopy (TEM) enables superior characterization of cell types through the analysis of their internal ultrastructures. In the fluid of *M. glacialis*, we identified (*via* TEM analyses) two main cell types of free-wandering coelomocytes that we assigned to the P2 population. The first coelomocyte type (**Figure 6A**) displayed an irregular shape, with numerous long, thin cytoplasmic processes. These cells averaged about 5-7 μm in diameter. We observed a nucleus size of 2-3 μm for cells with diameters in the upper range, these nuclei often had irregular shapes and were mainly euchromatic, with heterochromatin concentrated as scattered spots in the periphery. They often presented cell processes forming large loops which are typical of the inactive form of the known petaloid sub-population of coelomocytes (**Figure 6A**). The filopodial “activated” form was mainly found in coelomocyte clots/aggregates, where we observed increases in both the number and length of cytoplasmic processes. The cytoplasm was filled with numerous electron-lucent vesicles of varying sizes: most appeared optically empty though a few contained a fine granular material (**Figure 6A**). Only seldomly did we observe small phagosomes in the cytoplasm.

The second coelomocyte type also presented heterogeneity and shape irregularity (**Figure 6B**) as well as a similar cell body and nucleus size. These cells usually bore relatively few, and quite short cytoplasmic extensions, resembling pseudopodia. The cytoplasm was clearly less electron-dense than that described for the petaloid/filopodial cytotype and could contain numerous phagosomes, different inclusions that were heterogeneous in size and appearance (small, oval-shaped, electron-dense inclusions and a few small roundish, electron-lucent vesicles), and numerous mitochondria. Compared to the petaloid/filopodial cells, these exhibited much less electron-transparent vesicles, often were located in the peripheral cytoplasm, and presented a well-developed Golgi apparatus. Their ultrastructure visibly differed from that of the petaloid/filopodial cytotype previously described. However, as with the petaloid/filopodial cytotype, they underwent morphological transition (longer pseudopodia, irregular cell and nuclear shape, and a higher number of phagosomes) when activated.

Both coelomocyte types described here could be found free-wandering in the coelomic fluid or within aggregates (**Figure 6D**). In aggregates, they are mainly presented in their active forms, which are particularly evident in the coelomic fluid of regenerating starfish (*personal observation*). Heterogeneous aggregates of activated coelomocytes resembled clots formed by these cytotypes in the wounded area 24 hours following arm amputation, as we had previously observed in the same starfish species (not shown). These aggregates were also observed in our FC experiments (**Figure S1**).

Interestingly, among the free-wandering coelomocytes, we have also observed the sporadic presence of a third cytotype, possibly corresponding to the P1 cells. This cytotype presented a more undifferentiated aspect, with a cell body measuring 2-3 μm in diameter, a high nucleus/cytoplasm ratio and, occasionally, a few short processes branching outward that resembled those found in petaloid/filopodial cells (**Figure 6C**). We observed the nucleus to be mostly euchromatic, with an average diameter of around 1.5 μm and limited numbers of electron-lucent vesicles again, similar to those found in thrombocyte-like petaloid/filopodial cells sometime present in the cytoplasm. The cytoplasm staining and general appearance of this latter cell type resembled the clotting coelomocytes previously described.

## Discussion

In our study, we investigated new characterizations of the cell types present in the coelomic fluid of the asteroid *Marthasterias glacialis* with the main goal of better understanding their morphological heterogeneity and their specific physiological properties. We employed a novel combination of technologies that allowed us not only to effectively characterize those cell types but also to observe morphotypes that had not yet been described in the literature. Below we discuss the different findings and approaches.

### FC Fractionation Patterns Show Phagocytes as the Main Coelomocyte Population

FC fractionation of cell types in fluids is regularly performed in many species, although its use has been limited in members of the Echinodermata. In a previous study with FC done in the asteroid *Leptasterias polaris* only a single population of coelomocytes was observed (28). In specimens of *Asterias rubens* injected with artificial sea water, a main cluster, attributed to amoebocytes, was originally described, which was further sub-divided in three cellular groups that were differentially affected by bacterial challenge (29). FC profiles similar to ours were found by Xing et al. (30) for the circulating cells of the sea cucumber *Apostichopus japonicus* (Holothuroidea) and by Romero et al. (31) in the sea urchin *Paracentrotus lividus* (Echinoidea). These simple cytometric patterns are consistent with several reports (12, 20) that detected phagocytes as the most abundant, or even the only circulating coelomocyte type present in asteroids. Greater cellular diversity was described later by Smith et al. (32) in the echinoid *Strongylocentrotus purpuratus*. In their study, they used FC to select live cells based on exclusion of propidium iodide. These authors were able to discriminate among five populations of coelomocytes (based on their side- and forward scattering patterns). Phagocytes (or phagocyte-like cells) in this case were a heterogeneous population that contributed up to 70% of the circulating cells.

Direct comparison of echinoid coelomocyte types identified by FC with those of Asteroidea is a difficult task, given the previously described variability in coelomocyte types among echinoderm classes (7, 23) and diversity of protocols used in their isolation (33).

### Do Coelomocytes Have Diverse Ploidies?

Ploidy variation in an organism can be a defining feature of either pathological or normal developmental processes, while also contributing to differences between tissue or cell types in many species. So far, among 13 starfish species belonging to four families, Saotome et al. (34) reported that the genome is packed into 44 diploid chromosomes (34). A similar study carried out by Fafandel et al. (35) also reported the existence of two populations with diverse ploidy, in which the DNA content ratio between them was around 4:1. The authors suggested that some cells in the high-DNA population had probably engulfed other, unknown, nuclei. Although this type of situation was only reported during cell apoptosis (36), this result fits with the presence in the samples of haploid and diploid cells, as we suggest for some P1 morphotypes and for P2 cells, respectively. Interestingly, Fafandel et al. (35) reported that in the red starfish *Echinaster sepositus* circulating coelomocytes do not go through mitotic divisions. Rather, they suggested that all coelomocytes originated from the coelomic epithelium and are post-mitotic once they enter in the fluid. Their results contradict ours, although it should be considered that the DNA intercalator used in these previous studies (DAPI) stains only (or mostly) dead cells. This is the reason why in our assays we decided to use PI instead, which binds DNA more extensively either in dead or alive cells.

### Coelomocytes Present a Variety of Morphotypes

Brightfield and fluorescence microscopy have long been the methods of choice for the classification of echinoderm coelomocytes, especially when the process relies heavily on morphological characteristics (3, 6, 21, 37). Today, we understand that relevant differences exist among coelomocytes of different echinoderm classes, indeed, some morphotypes are not even represented in samples from members of the same species (3, 38). Again, disparity among protocols used for coelomic fluid collection, coelomocyte harvesting, staining conditions, sample preparation for microscopy, acquisition settings and image processing together with their reduced viability after collection and lack of resilience under manipulation contribute to increasing the range of detected (or missed) morphological complexity.

The two P1 morphotypes presented here, as detected by fluorescence microscopy, were also described in *A. amurensis* (39) and in *A. rubens* after staining with azure-eosin (21). In both studies these cells were detected in the coelomic fluid or included in the coelomic epithelium. Based on this observation, we would like to suggest that the P1L cell type corresponds to a non-differentiated form of the more mature P1S cell type. In line with our proposal, cells with P1L morphologies have been suggested to be the progenitor of the circulating cells (15); indeed, they displayed no overt signs of differentiation and constituted 50% of the weakly attached CE cells (21). Both CE cell types showed incorporation of BrdU, although in the coelomic fluid only the cells with smaller nucleus/cytoplasm ratio presented the same BrdU-positive feature (21). P1S properties such as proliferative activity, undifferentiated cell morphotype, ability to migrate and presence of euchromatin are also exhibited by other animals’ stem cells [*i.e.* planarian neoblasts (40)].

Our own results are consistent with the observations of Coteur et al. (29), which, aside from some dissimilarities in the FACS profiles, point to the presence in the coelomic fluid of a cell type with lower size and complexity (P1) in addition to the three morphotypes of larger cells (P2; regular, petaloid and filopodial). Using the same experimental conditions, the larger and more abundant coelomocytes (the P2 population) were classified as phagocytes by other authors (3). The higher forward scatter determined for P2 cells (see above), compared to P1, is justified by a more complex internal cellular architecture. According to Sharlaimova et al. (21, 39) P2 cells readily incorporate BrdU suggesting their proliferative activity *in vivo*. This observation agrees with our results on cell cycle analysis. Despite the similar cell-size distribution and similar nucleus cytoplasm diameter ratio, we observed morphological heterogeneity within this population, as described for echinoid coelomocytes (10). The petaloid form, named due to the presence of three-dimensional loops and convoluted plasma membrane folding, undergoes a rapid conversion to the filopodial form when activated by the contact with foreign particles, through manipulation or simply as the result of contact with air (6). In fact, Chia and Xing (41) reported that the transformation from the petaloid to the filopodial forms is associated with the activation of phagocytic processes in the sea cucumber *Holothuria leucospilota*. This change occurs *via* readjustments of the actin cytoskeleton (6, 18). Henson et al. (42) showed that the inhibition of arp 2/3 complex, the actin filament nucleator and branch inducer, in suspended echinoid coelomocytes drove a lamellipodial-to-filopodial shape change, and led to generation of a new structural organization of actin during cell spreading.

Also, from an ultrastructural point of view, our results are well corroborated by others studies in the literature. Large P2 coelomocytes often present cell processes forming large loops typical of the inactive form of the known petaloid sub-population of coelomocytes (3, 7, 41). For instance, Gorshkov et al. (18) also described a higher number and length of cytoplasmic processes in the filopodial form, very similar to those found in the free “mature coelomocytes” of *A. rubens*. Therefore, following our TEM results, the petaloid/filopodial coelomocytes can be consistently described as two functional states of a single cell type one which is actually specialized in clotting processes rather than participating in phagocytic activities. In vertebrates, the hemostatic function is performed by highly specialized cells as thrombocytes/platelets, which, once activated, undergo similarly drastic shape changes, including the extension of numerous filopodial processes (43, 44), as this occurs during the petaloid-filopodial transition of coelomocytes. These long, thin projections function to increase contact with other cells and create a clot. Therefore, we consider this latter cell morphotype to be functionally and structurally analogous to the thrombocyte-like cells of vertebrates. According to Levin (45), the first cells specialized in hemostasis probably appeared in non-mammalian vertebrates. Our results, however, open the intriguing possibility that this specialization in fact occurred (or even originated) at the base of the deuterostome lineage.

Considering their ultrastructural features, another P2 morphotype seems better fitted for performing phagocytic activities, therefore representing the functional equivalent of the vertebrate monocytes/macrophages. The activation of these phagocytes/immunocytes is essential for the inflammatory and immune responses triggered during wound healing, as is known to be true for all metazoan macrophages (46). In fact, the activated macrophages, not only phagocytize foreign material and cell debris, but also secrete a number of factors, such as pro-inflammatory cytokines and growth factors including TGF-β (47).

Smith et al. (3), reported that phagocytes tend to aggregate in clots during wound healing, particularly clots in which other coelomocytes are either trapped or actively contributing to their formation (24). These heterogenous aggregates of coelomocytes were also observed in the present study through the use of FC, IFC, fluorescence microscopy and TEM. The filopodial coelomocytes were generally detected at the periphery of the aggregate, whereas the macrophage-like cells were detected in the inner part, thus reaffirming the hemostatic role of the former. Furthermore, Ben Khadra et al. (9) already described a net-shaped syncytium of phagocytes covering the wound area of an amputated arm in the related starfish species *E. sepositus*.

## Conclusions

The present study constitutes the first attempt at cataloguing all cell types present in the coelomic fluid of the asteroid *M. glacialis*, native to the eastern Atlantic Ocean, using an innovative integrated classification approach that combines different methodologies: Flow Cytometry, Imaging Flow Cytometry, Fluorescence Microscopy and Transmission Electron Microscopy. The results of this work suggest that *M. glacialis* coelomic fluid is constituted by two different coelomocyte populations, here named P1 and P2, which have different cellular sizes and morphologies, and that are present at different relative abundances. The detailed study of cellular properties shows that some P1 morphotypes and P2 cells differ in ploidy and viability, as well as in cell-cycle parameters. Based on light microscopy observations, the most abundant cell population, P2, has a similar appearance to the already described echinoderm phagocytes, including the three known morphotypes (regular, filopodial and petaloid). P1 includes the smallest group of cells present in the coelomic fluid and exists as two differentiable morphotypes. From flow cytometry analysis, which allows us to quantify the relative abundance, cell viability and cell cycle distribution at the population level, we estimate the P2 population to represent 60-70% of total coelomocytes. P1 cells are able to incorporate exclusion dyes, right after their extraction from the organism, indicating that they have a low *ex vivo* viability.

In the recent literature, it is stated that 95% of starfish coelomocytes present clotting and phagocytic activity and are all classified as a single population called phagocytes/amoebocytes (3). From an ultrastructural point of view, our results confirm that the abundant P2 population can be actually ascribed to two distinct morpho-functional cytotypes, differentially involved in clotting phenomena or phagocytosis. These cells tend to aggregate, namely after clotting induction. Additionally, two other morphotypes were only detected under fluorescence microscopy and a third only when using IFC, revealing the importance of combining various approaches to avoid exclusion of morphotypes during CF cellular composition classification. Based mainly on results relating to ploidy, cell cycle and morphology, we suggest that the several P2 cytotypes described above represent morphologically and functionally differentiated sub-populations, possibly originating from a P1 progenitor pool. These undifferentiated cells could correspond to the undifferentiated “lymphocytes” often described in echinoderms (3) and could be the progenitors of at least this coelomocyte sub-population, as suggested for the undifferentiated cells of *A. rubens* (48), or even be *bona fide* stem cells with a relevant role in starfish regeneration processes. This hypothesis is in accordance with Gorshkov et al. (18) who also proposed that some cell morphotypes could correspond to diverse differentiation or activation stages.

Our study points to the need for further systematic characterization of the histology/physiology of these cell morphotypes, which in turn should be instrumental in ascertaining the origin and lineage relationships of those cells. The novel integrative FC/IFC/fluorescence microscopy/TEM methodology implemented in the present study could serve as a standard protocol for the classification of echinoderm coelomocytes populations. Moreover, we envision that our new experimental approach, with the aid of single cell sequencing methodologies, would be especially useful in the future elucidation of coelomocyte functional competences and developmental trajectories.

## Data Availability Statement

The original contributions presented in the study are included in the article/**Supplementary Material**. Further inquiries can be directed to the corresponding author.

## Author Contributions

AVC, MS, RG, and RZ developed the experimental design. BO, CB, and CA conducted the flow cytometry experiments, supervised by RG. BO, CA, JR, and AVC were involved in imaging flow cytometry experimental work. SG, CF, and MS performed the electron microscopy assays. BS and AVC performed the fluorescence microscopy experiments. All authors contributed to the analysis of the experimental results and to writing the manuscript. The whole project was coordinated by AVC. All authors contributed to the article and approved the submitted version.

## Funding

To the “Maristem COST Action” (CA16203), supported by COST (European Cooperation in Science and Technology), for funding PM STSM visits (February 2019 and November 2020) to the AVC Laboratory.

## Conflict of Interest

The authors declare that the research was conducted in the absence of any commercial or financial relationships that could be construed as a potential conflict of interest.
