# Supplementary material for: Characterization of Coelomic Fluid Cell Types in the Starfish Marthasterias glacialis Using a Flow Cytometry/Imaging Combined Approach
Source: Front Immunol. 2021 Mar 18;12:641664. doi: 10.3389/fimmu.2021.641664 (PMC8013778; doi:10.3389/fimmu.2021.641664)
Supplement: Supplementary file 1 [file DataSheet_1.docx]

**
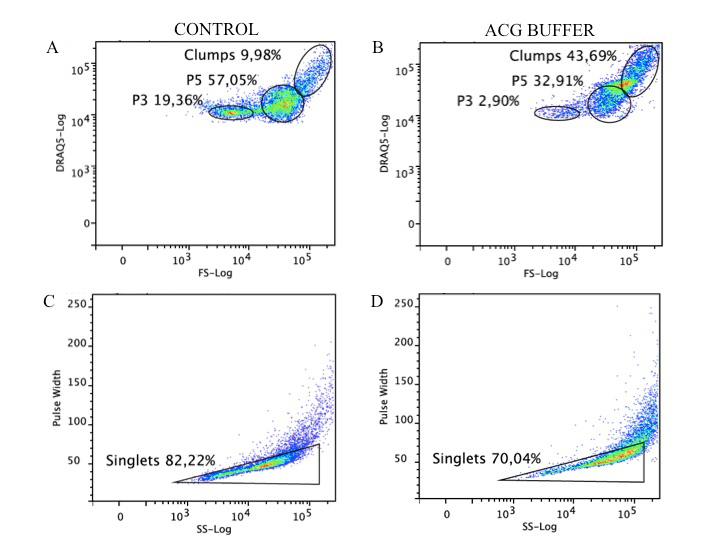
**

**Singlets 70%**

**Singlets 82%**

**P1 3%**

**P2 33%**

**Aggregates 44%**

**Aggregates 10%**

**P2 57%**

**P1 19%**

**Figure S1 Flow cytometric analysis of circulating coelomocytes using the anticoagulant buffer** (0.5 M NaCl, 5 mM MgCl_2_, 20 mM HEPES and 1 mM EGTA pH=7.5**).** Coelomocytes were stained with DRAQ5. **A, C.** Dot plots representation of **(A)** coelomocyte populations and **(C)** its singlets gated without using anticoagulant solution. **B, D.** Dot plots representation of anticoagulant solution effect in **(B)** coelomocyte populations and **(D)** its singlets gated.
