# Supplementary material for: Characterization of Coelomic Fluid Cell Types in the Starfish Marthasterias glacialis Using a Flow Cytometry/Imaging Combined Approach
Source: Front Immunol. 2021 Mar 18;12:641664. doi: 10.3389/fimmu.2021.641664 (PMC8013778; doi:10.3389/fimmu.2021.641664)
Supplement: Supplementary file 2 [file Table_1.docx]

**Table S1 Relative abundance of Flow Cytometry populations**

| Biological Replicates | P1(%) | P2(%) |
| --- | --- | --- |
| 1 | 3.0 | 65.4 |
| 2 | 4.4 | 60.5 |
| 3 | 11.0 | 65.3 |
| 4 | 9.8 | 61.8 |
| 5 | 10.5 | 63.4 |
| 6 | 7.6 | 63.7 |
| Average | 7,7 | 63,4 |
